# Cytomegalovirus in pregnancy: to screen or not to screen

**DOI:** 10.1186/1471-2393-13-96

**Published:** 2013-04-18

**Authors:** Susan P Walker, Ricardo Palma-Dias, Erica M Wood, Paul Shekleton, Michelle L Giles

**Affiliations:** 1 Department of Perinatal Medicine, Mercy Hospital for Women, 163 Studley Road, Heidelberg, VIC 3084, Australia; 2 Department of Obstetrics and Gynaecology, University of Melbourne, Melbourne, VIC, Australia; 3 Department of Fetal Medicine Unit, Melbourne, VIC, Australia; 4 Department of Infectious Diseases, the Royal Women's Hospital, Melbourne, VIC, Australia; 5 Departments of Clincial Haematology, Royal Women’s Hospital, Melbourne, VIC, Australia; 6 Department of Infectious Diseases, Monash Health, Melbourne, VIC, Australia; 7 Department of Haematology, Monash University, Melbourne, VIC, Australia; 8 Department of Fetal Diagnostic Unit, Melbourne, VIC, Australia; 9 Department of Infectious Diseases, Monash University, Melbourne, VIC, Australia

## Abstract

**Background:**

Cytomegalovirus (CMV) infection is now the commonest congenital form of infective neurological handicap, recognized by the Institute of Medicine as the leading priority for the developed world in congenital infection. In the absence of an effective vaccine, universal screening for CMV in pregnancy has been proposed, in order that primary infection could be diagnosed and- potentially- the burden of disability due to congenital CMV prevented.

**Discussion:**

Universal screening for CMV to identify seronegative women at the beginning of pregnancy could potentially reduce the burden of congenital CMV in one of three ways. The risk of acquiring the infection during pregnancy has been shown to be reduced by institution of simple hygiene measures (primary prevention). Among women who seroconvert during pregnancy, CMV hyperimmune globulin (CMV HIG) shows promise in reducing the risk of perinatal transmission (secondary prevention), and CMV HIG and/ or antivirals may be effective in reducing the risk of clinical sequelae among those known to be infected (tertiary prevention). The reports from these studies have re-ignited interest in universal screening for CMV, but against the potential benefit of these exciting therapies needs to be weighed the challenges associated with the implementation of any universal screening in pregnancy. These include; the optimal test, and timing of screening, to maximize detection; an approach to the management of equivocal results, and the cost effectiveness of the proposed screening program. In this article, we provide an overview of current knowledge and ongoing trials in the prevention, diagnosis and management of congenital CMV. Recognising that CMV screening is already being offered to many patients on an *ad hoc* basis, we also provide a management algorithm to guide clinicians and assist in counseling patients.

**Summary:**

We suggest that- on the basis of current data- the criteria necessary to recommend universal screening for CMV are not yet met, but this position is likely to change if trials currently underway confirm that CMV HIG and/ or antivirals are effective in reducing the burden of congenital CMV disease.

## Background

Cytomegalovirus (CMV) infection remains the commonest cause of infective neurological handicap since implementation of universal rubella vaccination. The number of children affected by congenital CMV is similar to other conditions such as Down Syndrome, for which routine screening is advocated and community awareness is high [1]. The birth prevalence of congenital CMV is estimated at 0.64% and 11% of these infants are symptomatic [2]. This equates to a birth prevalence of approximately 7/10,000 affected infants, not dissimilar to conditions for which screening is currently recommended, such as early onset groups B streptococcus infection, with a prevalence of 4.3/10,000 [3] and Down syndrome with a birth prevalence of 11/10,000 births [4]. CMV infected infants who are symptomatic at birth have a 5-10% neonatal mortality rate and, among survivors, sequelae may be severe and lifelong [5]. CMV may also be an important contributor to antenatal stillbirth [6]. Accordingly, the Institute of Medicine has identified development of a CMV vaccine as the highest priority in congenital infectious diseases in the developed world but, while results from a recent phase 2 vaccine trial are encouraging [7], there is no effective vaccination imminent.

### The potential role of screening

In an attempt to reduce the disease burden of congenital CMV, some clinicians and patient groups have advocated for CMV screening in pregnancy in order that primary infection- that associated with the highest risk of both perinatal transmission and clinical consequences- can be diagnosed and, potentially, congenital CMV and its sequelae prevented. Screening for CMV could take one of several forms. The *first approach* would be universal screening of all women prior to, or in early pregnancy, to (i) identify seropositive women who could be reassured that they are not at risk of primary CMV in pregnancy, and (ii) identify seronegative women who can be given advice to minimize CMV acquisition in pregnancy. Such women may be offered serial serology during pregnancy, to look for evidence of serconversion. The *second approach* is to only screen women at increased risk. The highest risk group comprises women with frequent or prolonged contact with children under the age of three; women with young children at home or those that work in a day care setting. A *third approach* is to perform ‘once off’ serology, including avidity testing at around 20 weeks in order to identify most primary infections that have occurred early in pregnancy (the time of greatest risk). The *final screening approach* is that most aligned with current clinical practice; targeted assessment on the midtrimester morphology ultrasound for features of congenital CMV (such as ventriculomegaly, intracerebral calcifications, microcephaly, echogenic bowel, midtrimester intra-uterine growth restriction), and secondary maternal serology screening if positive features are identified. Each method has obvious benefits and limitations. Cahill et al have performed a cost-effectiveness analysis, modeling the latter three strategies, and concluded that universal screening is the most cost-effective approach, assuming an efficacious treatment in the form of CMV hyperimmune globulin (discussed below) was available [8]. Nevertheless, the proposed ‘once off serology’ has some limitations; seronegative women are unable to be advised on strategies to minimize CMV acquisition in early pregnancy; some women will have been exposed but not yet seroconverted at the time of serology; some women who have had periconceptual or early pregnancy infection may have avidity above the threshold; and the opportunity to reduce fetal infection with treatment in pregnancy may have been lost. It is for these reasons that several countries have adopted early or pre pregnancy screening, followed by serial follow up serology of seronegative women in order to detect seroconversion. The remainder of this article addresses how such an approach might work in clinical practice.

### Potential interventions

Our advancing knowledge of CMV means that many of the Wilson and Junger criteria commonly applied to screening tests [9] are now met for CMV; CMV is an important condition, diagnostic testing is available and the natural history of the condition is known. Nevertheless, universal screening for CMV would be predicated on the assumption that a safe, cost effective and accepted treatment or intervention exists to reduce the risk of congenital CMV among women found to be seronegative. Universal screening in pregnancy is offered for conditions of even lower prevalence such as Rubella [10], Hepatitis B [11] and syphilis [12] because of the opportunity to reduce congenital infection with simple, safe and effective interventions such as post partum rubella vaccination, post exposure prophylaxis for Hepatitis B, and timely treatment of maternal syphilis with penicillin. Interventions to reduce the risk of congenital CMV among seronegative women may be one of three types; *primary prevention* (preventing maternal seroconversion during pregnancy), *secondary prevention* (preventing fetal transmission following maternal seroconversion) or *tertiary prevention* (preventing disease sequelae among infected fetuses). Many of the knowledge gaps surrounding such interventions are beginning to close, re-igniting the screening debate. In part this is because several countries, including Israel and some European centres, have been conducting population based studies incorporating universal screening for some time. From such studies has come valuable information on CMV epidemiology [13,14], reliability of serological screening [15], key biological mechanisms for placental and fetal damage [16] and the potential efficacy of prevention strategies [17] and fetal treatment [18]. These findings highlight the value of screening studies not just to the individual, but to the broader medical community, providing the necessary data to inform future screening policy.

**Figure 1:**
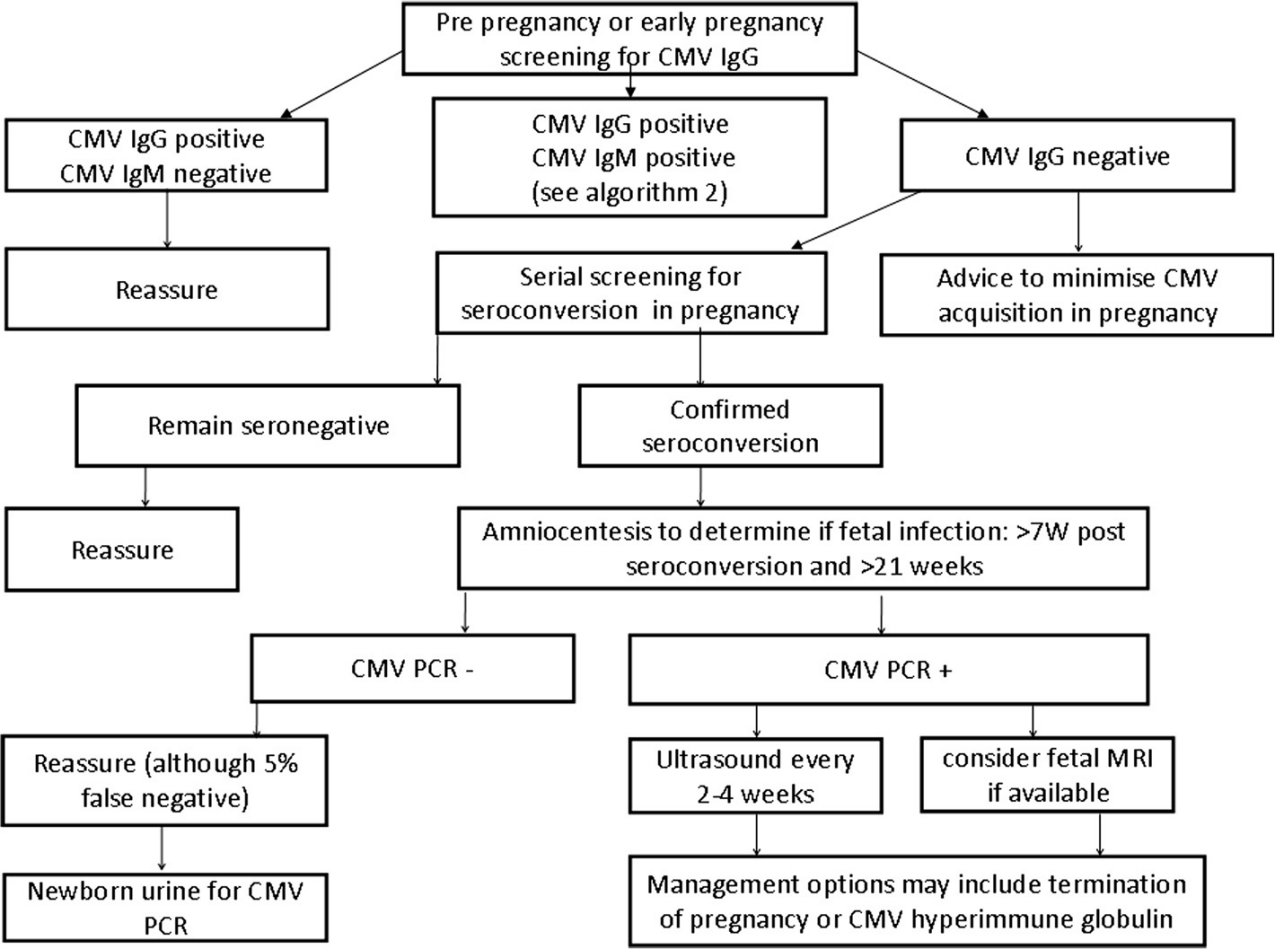
Proposed algorithm for CMV screening.

**Figure 2:**
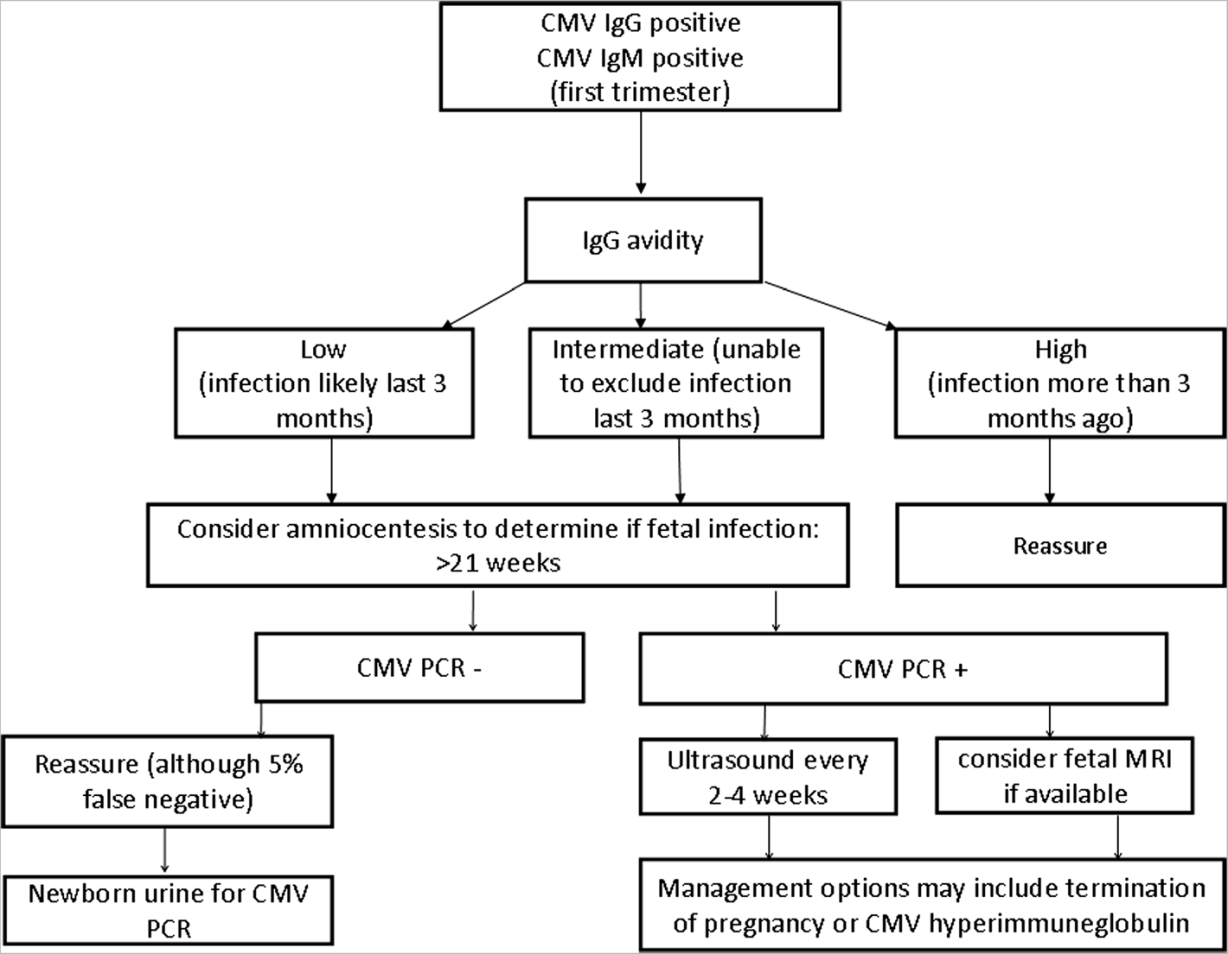
Proposed algortithm for management of women with IgG positive/IgM positive results at the time of screening.

In this article we provide a review of these and other studies, summarizing current knowledge and ongoing trials in the prevention, diagnosis and management of congenital CMV. Recognising that many clinicians have already commenced screening using one or more of the proposed screening strategies, we present the evidence in a temporal clinical sequence; (i) the management of seronegative women; (ii) the management following maternal seroconversion and (iii) the management of pregnancies where the fetus is known to be infected, summarized in Figures 1 and 2.

## Discussion

### Management of seronegative women

Whilst it is recognized that infants can have congenital CMV as a result of maternal CMV re-infection or reactivation during pregnancy [19] (so called non primary infections), the risk of perinatal transmission [2] and subsequent sequelae involving long-term disability [5] is much higher among primary than non-primary infections. Accordingly, prevention of primary CMV during pregnancy is recognized as being of the greatest importance. Among women, seroprevalence increases from 56% between the ages of 30-34 years to 79% between the ages of 35-39 years, which is consistent with other studies suggesting approximately 50% of pregnant women are CMV non-immune. Of these, 1-2% will undergo seroconversion during pregnancy [19]. High-risk populations for seroconversion include day care workers (8% seroconversion per annum), and parents of children shedding CMV (24% seroconversion per annum) [20]. Seroconversion may be silent as CMV infection in immunocompetent adults is commonly asymptomatic, although a retrospective history of symptoms (flu like symptoms, fever, myalgia) and accompanying laboratory features (lymphocytosis, elevated amino transferase) was reported in over 50% of pregnant women with primary CMV in one study [21]. Universal screening for seroconversion is nevertheless the most reliable means of identifying primary infection in pregnancy.

#### Hygiene advice to seronegative women

One of the benefits of universal screening is that seronegative women can be identified and advised how to minimise the risk of seroconversion during pregnancy. A summary of the measures recommended by the Centres for Disease Control and Prevention are presented below [22].Such measures have been proven to be effective. A French study reported on over 2,500 CMV seronegative women at 12 weeks gestation. Institution of simple hygiene measures such as regular hand washing, and avoiding intimate contact with children under the age of 6 years, resulted in rate of serocoversion prior to 36 weeks gestation of only 0.2% among these women [17]. In a related study, this group reported that CMV screening was received favourably when offered to women at 12 weeks’ gestation [23]. In this study, 3792 women known to be non-immune, or with unknown immune status, were advised of the potential consequences of primary CMV, were given hygiene advice to minimise CMV acquisition, and were advised regarding the paucity of scientific data on the value of screening. Despite this, 96.7% of women accepted serological screening, suggesting that the uptake of CMV screening when women are informed of the importance of CMV infection, is likely to be high.

•Wash hands with soap and water for 15-20 seconds after handling body fluids, dirty laundry or children’s toys, changing nappies, bathing or feeding young children

•Do not share food, drinks or eating utensils with young children; do not put a dummy in your mouth or share a toothbrush with a young child

•Avoid contact with saliva when kissing a child

•Clean toys, countertops etc that come into contact with children’s urine or saliva

#### Diagnosis of primary infection

Seroconversion can be reliably diagnosed when a previously seronegative woman develops CMV-specific IgG and IgM antibodies. In the absence of previous serology, CMV primary infection may be suspected in the presence of IgM and low avidity IgG. CMV IgM positivity does not always reflect recent infection and may be either a false positive (related to intercurrent viral infection or presence of auto-immune disease) or be a prolonged IgM response (IgM seropositivity may persist for months to years). In this scenario, IgG avidity may help to distinguish recent from remote infection. Avidity refers to the strength of binding of an antibody to the viral epitope, reflecting the maturity of the antibody response. While the cut-off for high avidity will vary according to the assay used, and there are variations in results between assays, the presence of a high avidity IgG generally infers that primary infection occurred ≥12-16 weeks previously [15]. Expert interpretation of serology in the setting of a CMV IgM positive result is important to ascertain whether this is as a result of a primary, non primary or past infection. Given the lower incidence of transmission and sequelae, confirmation of past or non primary infection will provide reassurance to many women, evidenced by the reduced incidence of invasive testing and termination of pregnancy in this group [24]. If primary infection is confirmed, it is important that expert advice is sought from a maternal fetal medicine and/or infectious diseases specialist to provide advice on diagnosing fetal infection, predicting infant sequelae and the emerging role of in utero therapy.

### Management following maternal seroconversion

#### Diagnosing fetal infection

The risk of fetal infection following primary maternal seroconversion is estimated at 32.3% overall, much higher than the risk associated with non primary infection (1.4%) [2]. The risk of fetal infection is gestation dependent; a recent series of 248 cases of primary CMV confirmed a fetal transmission rate of 17% when maternal infection occurred preconceptually (from 1-10 weeks prior to last missed period), 35% when infection occurred periconceptually (defined as 1 week prior to 5 weeks post missed period), and 30%, 38% and 72% for the remainder of the first trimester and second and third trimesters, respectively [14]. These transmission rates are remarkably similar to those reported in a Belgian study in 2010, the largest study to date. Among 524 seroconversions, the mean rate of transmission was 47%, rising from 35% where seroconversion occurred in the first trimester to 44% and 73% when seroconversion occurred during the second and third trimester, respectively [13].

Confirmation of fetal infection requires an amniocentesis. In order to allow sufficient time for placental transmission, renal infection and tubular excretion, establishment of adequate diuresis and excretion of CMV into the fetal urine, amniocentesis has maximal sensitivity if performed 7 weeks after maternal seroconversion and should be performed after 20 weeks gestation, whichever is later [25]. A positive result for CMV using PCR on the amniotic fluid confirms fetal infection (100% positive predictive value), but is not predictive of long-term sequelae. The negative predictive value of amniocentesis for fetal infection is approximately 95% [26]. In a series of 10 congenitally infected infants whose amniocenteses were negative at 21 weeks (performed at least 7 weeks after presumed maternal infection), four had CMV-related morbidity diagnosed at follow up (sensorineural hearing loss), two of whom responded to antiviral treatment [27]. This underscores the importance of newborn screening by way of a neonatal urine in the first three weeks of life for all children born to women who have seroconverted in pregnancy, including those with a negative PCR on amniocentesis.

#### Can fetal infection be prevented?

In 2005, a non-randomised study reported a reduction in vertical transmission from 40% (19/47) to 16% (6/37) among women with CMV seroconversion in pregnancy following maternal administration of CMV hyperimmune globulin (CMV HIG) [18]. Intravenous immunoglobulin (IVIG) was first used in the 1950s, and is a concentrated plasma product containing at least 90% intact IgG, generated from a pool of screened plasma donors. IVIG has a favourable safety profile in pregnancy, with adverse reactions occurring in less than 5% of patients. IVIG has been safely used for immunomodulation in pregnancy for conditions such as autoimmune and allo-immune thrombocytopenia and severe red blood cell iso-immunisation since the 1980s [28]. CMV HIG is collected only from donors with high levels of CMV IgG. The reduction in fetal transmission among women with primary CMV in pregnancy is presumed attributable to the high IgG avidity in the CMV HIG, which has both neutralising and immunomodulating activity. Buxman et al have also reported lower than expected rates of CMV transmission (23%) following administration of CMV HIG to women with a periconceptual, first or second trimester diagnosis of primary CMV [29].

The data from these two small studies are encouraging, and three large randomised controlled trials have commenced examining the utility of administering CMV HIG to pregnant women who have seroconverted during pregnancy to prevent fetal infection [30,31]. The efficacy study of Human CMV (HCMV) hyperimmune globulin to prevent congenital HCMV infection (CHIP) trial has completed and preliminary results have been presented [32]. In summary, the trial included 122 women with primary CMV confirmed between 5 and 26 weeks gestation, where randomisation and treatment were required within 6 weeks of the presumed onset of maternal infection. The trial was powered to show the same magnitude of reduction in transmission with CMV HIG administration as was seen in the original Nigro trial (ie from 40% to 16%) [18]. This trial reported a trend toward reduced transmission among those administered CMV HIG, from 44% to 32%, although this result failed to achieve statistical significance. The remaining two prevention trials, one based in Europe [31] and the other based in the United States are yet to complete recruitment.

Based on the currently available evidence, the two studies to date- if combined – would suggest an approximate reduction in fetal transmission of 50% with timely administration of CMV HIG following maternal seroconversion, but results of the ongoing trials will be able to estimate this effect with greater precision, and will be critical in determining the place of CMV screening in pregnancy. These prevention trials will also be crucial in determining the optimal rescreening interval of seronegative women during pregnancy. If administration of CMV HIG is confirmed to significantly reduce the incidence of fetal infection following maternal seroconversion, then repeat serological testing at frequent intervals during pregnancy would be indicated to optimise timely detection of primary CMV, and enable prompt institution of treatment. Monthly rescreening has been has been reported in some series [18], which may optimise detection of maternal seroconversion and reduce the risk of fetal infection prior to CMV HIG administration, but the feasibility and cost effectiveness of such an approach on a large scale remains to be determined.

### Management of pregnancies where the fetus is known to be infected

#### The risk of fetal sequelae

Clinical sequelae resulting from CMV infection may result from placental and/or fetal infection. Histological changes associated with placental CMV infection include avascular and hydropic villi, fibrinoid deposits and syncytial knotting [16], all of which may impair transport of oxygen and nutrition to the developing fetus. Clinical sequelae from placental infection may include intra-uterine growth restriction, hepatosplenomegaly and stillbirth. Sequelae such as central nervous system damage and sensori-neural hearing loss result more directly from fetal cytopathic injury, although modulation of placental immune function may also play a role in mediating fetal injury [33]. The risk of long term complications is inversely related to gestational age, with the highest rate of severe sequelae associated with infection in the first trimester. Neurological sequelae (including hearing loss) have been reported in approximately 30% of infants with congenital CMV where maternal infection was diagnosed in the first trimester, which is more than twice the rate of sequelae following later infection [34]. Overall, 10-15% of congenitally infected infants will have symptoms at birth, including jaundice, hepatosplenomegaly, hydrops, thrombocytopenia, anaemia, microcephaly, seizures and chorioretinitis. Among these infants, perinatal mortality may be as high as 10%, and the risk of long-term neurological sequelae among survivors lies between 40% and 60% [5]. Of the remaining 90% of infants that are asymptomatic at birth, approximately 10-15% will develop symptoms later, most commonly sensorineural hearing loss [5].

#### Predicting symptomatic infants

In ongoing pregnancies where fetal infection has been confirmed on amniotic fluid, ultrasound surveillance is recommended for evolving features of CMV-related fetal damage, including the development of intra-uterine growth restriction, and oligo- or polyhydramnios. System specific features include the presence of echogenic bowel, intra-hepatic calcifications (intra-hepatic echodensities with brightness equivalent to bone and with posterior acoustic shadowing [35]), pleural effusions, ascites or generalised hydrops. The optimal frequency of ultrasound surveillance is unknown, but ultrasound examinations are generally performed every 2-4 weeks following confirmation of fetal infection.

Optimal evaluation of the central nervous system (CNS) with ultrasound involves targeted, serial ultrasound examination, often employing the transvaginal, approach to optimise CNS views. CNS findings associated with congenital CMV include ventriculomegaly, the presence of intra-ventricular adhesions, periventricular calcifications and cysts, cortical or cerebellar abnormalities and the presence of haemorrhage. Many studies have reported that ultrasound identifies only a minority of fetuses who will subsequently be found to be symptomatic at birth [36], but Farkas et al have recently reported in a small study that congenitally infected children with normal imaging showed no difference in cognitive, language or motor development when compared to gestation-matched controls at a median follow up of 34 months [37]. This small study suggests that, to optimise sensitivity, follow up examinations of congenitally-infected fetuses should be performed by those with expertise in obstetric neurosonography.

The role of fetal MRI is evolving in the evaluation of developmental or acquired brain lesions, with MRI providing superior assessment of the cerebral cortex and white matter. Among fetuses with confirmed CMV infection, the use of MRI in addition to ultrasound improves the positive predictive value for brain lesions confirmed on postnatal examination from 71% with ultrasound alone to 89% [37]. The sensitivity of fetal MRI increases with advancing gestation, although the upper gestational limit may be influenced by pragmatic considerations such as availability of *in utero* treatment and access to late termination of pregnancy in the event that significant abnormalities are confirmed.

Where significant CNS changes are observed on ultrasound (ventriculomegaly, microcephaly, cerebellar hypoplasia), it is highly likely that these will be associated with significant sequelae. Benoist et al has reported that a ‘symptomatic ultrasound’ was associated with an 85-90% risk of brain abnormalities being confirmed at post mortem (following termination of pregnancy) or postnatal ultrasound examination [38]. Recent studies suggest that the combination of expert neurosonography and MRI improves the detection of CNS abnormalities among CMV-infected fetuses, and that normal examinations provide some reassurance [39,40]. Nevertheless, not all sequelae are detectable with antenatal imaging, the most obvious being sensorineural hearing loss, and the limitations of imaging in predicting outcome need to be addressed during counselling.

#### Can in utero treatment reduce the risk of sequelae among infected infants?

In the past, the only effective means of preventing disability due to congenital CMV was to offer termination of pregnancy upon the antenatal diagnosis of definite or possible sequelae. The difficulties of this approach are compounded by the fact that by the time consequences of infection have become apparent on imaging, access to termination of pregnancy may be uncertain, with varying legislation on acceptable indications for, and upper gestational limit of, termination of pregnancy. Alternate management of the affected fetus includes in utero therapy with CMV HIG or antivirals, with recent studies reporting encouraging results [18,41].

One non-randomised clinical trial in 2005 suggested that CMV HIG may be efficacious in treating congenital CMV in utero, with reduced sequelae reported at 2 year follow up [18]. This encouraging early data has been followed by two further studies, both confirming fetal benefit with administration of CMV HIG following confirmation of fetal infection. Visentin et al in a non randomised study reported on the 12 month follow up of 68 infants confirmed to have fetal infection during pregnancy. Poor outcome at 12 months (defined as audiological or neurological abnormality, presence of necrotizing enterocolitis or chronic liver disease) was found in 16 of 37 untreated women (43%) compared to 4 of 31 (13%) of women treated with a single dose of CMV HIG between 20 and 24 weeks following the diagnosis of fetal infection (P < .01) [41]. In a recent case control study, Nigro et al confirmed that the only significant risk factor for having a symptomatic infant at 1-5 years of age following the diagnosis of congenital CMV at less than 20 weeks gestation was the mother not having received CMV HIG [42]. Such benefits are presumed due to both the high avidity and neutralizing activity of CMV HIG, and/or down-regulation of immune-mediated fetal and placental damage [16]. Administration of standard intravenous immunoglobulins (IVIG), obtained from an unselected donor pool, has also been demonstrated to increase CMV IgG and avidity among women with primary infection in pregnancy. If future studies demonstrate comparable efficacy, this may be able to reduce the cost and improve access to such therapy [43]. There are no ongoing trials specifically examining the role of CMV HIG for treatment of symptomatic disease in infected fetuses, and administration of CMV HIG to women known to have an infected fetus is becoming standard practice. Further data on the value of treatment is only likely to come from prospective registries comparing the outcomes of CMV HIG treated, versus untreated, pregnancies.

A single trial has evaluated the role of administering oral valacyclovir to women carrying a fetus with confirmed CMV infection. Although this trial demonstrated a significant reduction in fetal viremia post treatment with valacycolvir, the outcome among treated infants was no different to an untreated historical control group [44]. A double blind randomised controlled trial is currently underway to evaluate the role of valacyclovir in women whose fetuses demonstrate extracerebral manifestations of CMV infection; completion of this trial is expected in 2013.

## Summary

Congenital CMV is the commonest infective cause of neurological handicap and is a significant contributor to long term disability. Congenital CMV is common and important enough to justify its place among other routine antenatal screening tests. The case for CMV screening in pregnancy is supported by the proven reduction in maternal primary infection following institution of simple hygiene measures (primary prevention). On the other hand, further data is awaited from randomized trials currently underway to better estimate the reduction in fetal infection achieved with CMV HIG among recently seroconverted women (secondary prevention). In utero treatment with CMV HIG appears to reduce clinical sequelae among fetuses confirmed to be infected (tertiary prevention), and CMV HIG is currently being offered to women with a known infected fetus identified through current screening strategies. An ongoing trial will soon clarify the role of antivirals such as valacyclovir to reduce sequelae in symptomatic fetuses.

The results of these ongoing trials will inform the current clinical algorithm for diagnosis and management of maternal and fetal CMV infection in order that diagnostic tests and therapies may be appropriately targeted, and cost effectiveness of screening models can be established. Whether universal serological screening should be implemented for the detection of primary CMV in pregnancy in 2013 may be neither ‘*yes*’ nor ‘*no*’, but ‘*not yet*’, recognizing that this position may soon change if trials currently underway confirm that intervention in pregnancy can reduce the risk of fetal infection and, in turn, the considerable burden of death and survival with disability due to congenital CMV.

## Competing interests

The authors declare that they have no competing interests.

## Authors’ contributions

SW, RPD, EW, PS and MG all contributed to the conception of the paper, drafted and revised the manuscript, and all authors read and approved the final manuscript.

## Pre-publication history

The pre-publication history for this paper can be accessed here:

http://www.biomedcentral.com/1471-2393/13/96/prepub
